# MicroRNAs in Cancer Management: Big Challenges for Small Molecules

**DOI:** 10.1155/2015/982156

**Published:** 2015-03-24

**Authors:** Paolo Gandellini, Elisa Giovannetti, Francesco Nicassio

**Affiliations:** ^1^Department of Experimental Oncology and Molecular Medicine, Fondazione IRCCS Istituto Nazionale dei Tumori, Via Amadeo 42, 20133 Milan, Italy; ^2^Department of Medical Oncology, VU University Medical Center, De Boelelaan 1117, 1081 HV Amsterdam, Netherlands; ^3^Center for Genomic Science of IIT@SEMM at the Istituto Italiano di Tecnologia, Via Adamello 16, 20139 Milan, Italy

In the last decade, the scientific community has been shaken by what we call the “noncoding revolution.” We have witnessed the discovery of an increasing amount of RNA molecules, which play a critical role in normal physiology as well as disease, without providing any protein product. The smallest regulatory RNA species, known as the ~22 nucleotide-long microRNAs (miRNAs), were at the forefront of such a revolution.

According to the last release of miRNA database (*miRBase* http://www.mirbase.org—release 21) more than 35,000 miRNA species have been identified, of which a total of more than 2,500 mature miRNAs exist only in humans [1]. Since their first discovery in* C. elegans* in 1993, it has become clear that these tiny molecules have an enormous regulatory potential, being able to exert negative posttranscriptional regulation on hundreds of protein coding genes, even simultaneously, and ultimately act as master regulators of entire biological processes.

Besides their role in development, the involvement of miRNAs in human disease, and cancer in particular, has attracted major attention. In fact, deregulated miRNA expression could lead to aberrant expression of targeted oncogenes or tumor suppressors, thus resulting in tumor development and progression. Accordingly, altered expression of miRNAs has been observed in almost every tumor type, including haematological malignancies, carcinomas, sarcomas, and central nervous system neoplasms. Relevant examples of the role of miRNAs on specific cancer types are reported in this issue, with a focus on childhood acute lymphoblastic leukemia (original contribution by M. Duyu et al.), ovarian and cervical cancer (reviewed by Y. Kinose et al. and S. M. Díaz-González et al.), soft tissue sarcomas (reviewed by T. Fujiwara et al.), or medulloblastoma (reviewed by S. López-Ochoa et al.).

The unique pattern of altered miRNA expression provides a fingerprint that may serve for cancer diagnosis and prediction of patient's prognosis or response to treatment. In addition, a number of miRNAs have been shown to directly participate in tumorigenesis by acting as “oncoMirs” or “tumor suppressive miRNAs,” thus becoming potential key targets or tools for anticancer therapy. The review by A. Saumet and colleagues suitably introduces the current approaches and applications of miRNAs in cancer and human diseases, from expression analyses aimed at identifying miRNAs potentially useful for tumor diagnosis or prognosis to the potential use of them as novel therapeutic agents.

The opportunities and challenges of miRNAs as cancer biomarkers have been addressed in the issue by H. Lan and colleagues. Specific focuses have been also provided on the clinicopathological significance of miR-155 in breast cancer (H. Zeng et al.), diagnostic and prognostic miRNAs in sarcomas (T. Fujiwara et al.), and miRNAs exploitable for prostate cancer risk assessment (A. Cannistraci et al.). Based on their relative stability in body fluids, a great effort has been devoted in the last years to the study of circulating miRNAs as noninvasive biomarkers for tumor diagnosis or disease monitoring. Though attracting, miRNA quantification in liquid samples is far from being trivial. Challenges encountered in the field have been extensively addressed in the issue by P. Tiberio and colleagues.

Due to their nature as physiological molecules able to control multiple genes at the same time, miRNAs are also considered as very promising therapeutic targets or tools. As tumors typically evade cancer treatment by acquiring secondary mutations on targeted proteins, it appears more difficult for a tumor to escape from miRNA effects, which are directed on multiple proteins at the same time. Controlling the delivery and activity of miRNA-modulating agents into specific tissues or organs still appears as the “the big challenge,” albeit a number of promising approaches are under validation. This contention is extensively explored in this issue in different contexts. The review by O. Fortunato et al. focuses on lung cancer, one of the big cancer killers, and discusses the most likely miRNA targets as well as the methodological approaches for* in vivo* delivery of miRNAs. The use of miRNA modulators for a direct antitumor effect, when administered alone or in combination with chemo- or radiotherapy, has been described in the prostate cancer context by A. Cannistraci and colleagues. Furthermore, the use of miRNAs for indirect anticancer effects has been reviewed by S. Gallach et al., who discussed on the possibility to modulate miR-126 or miR-92a to target tumor angiogenesis, thus limiting tumor growth and metastatic spread, or act on hypoxia. Finally the role of miRNAs in determining and regulating chemoresistance of pancreatic cancer has been discussed by I. Garajová and colleagues.

Overall the issue is intended to provide evidence of the potential usefulness of miRNAs in cancer management as well as focus on the still unsolved technical and conceptual challenges in miRNA research.

*Paolo  Gandellini* *Elisa  Giovannetti* *Francesco  Nicassio*
